# Multistate Outbreak of *Salmonella* Infections Linked to Raw Turkey Products — United States, 2017–2019

**DOI:** 10.15585/mmwr.mm6846a1

**Published:** 2019-11-22

**Authors:** Rashida Hassan, Sean Buuck, Douglas Noveroske, Carlota Medus, Alida Sorenson, Jessica Laurent, David Rotstein, Linda Schlater, Jennifer Freiman, Aphrodite Douris, Mustafa Simmons, Danielle Donovan, Justin Henderson, Mackenzie Tewell, Kaitlyn Snyder, Oluwakemi Oni, Diana Von Stein, Kossia Dassie, Molly Leeper, Azizat Adediran, Natasha Dowell, Laura Gieraltowski, Colin Basler

**Affiliations:** ^1^Division of Foodborne, Waterborne, and Environmental Diseases, National Center for Emerging and Zoonotic Infectious Diseases, CDC; ^2^CAITTA, Inc., Herndon, Virginia; ^3^Minnesota Department of Health, Saint Paul, Minnesota; ^4^Food Safety and Inspection Service, U.S. Department of Agriculture, Washington, D.C. and Athens, Georgia; ^5^Minnesota Department of Agriculture, Saint Paul, Minnesota; ^6^Center for Veterinary Medicine, U.S. Food and Drug Administration, Rockville, Maryland; ^7^Animal and Plant Health Inspection Service, U.S. Department of Agriculture, Riverdale Park, Maryland; ^8^Michigan Department of Health and Human Services, Lansing, Michigan; ^9^Michigan Department of Agriculture and Rural Development, Lansing, Michigan; ^10^Arizona Department of Health Services, Phoenix, Arizona; ^11^Iowa Department of Public Health, Des Moines, Iowa; ^12^DC Health, Washington, D.C.; ^13^Eagle Medical Services, Huntsville, Alabama.

During 2018–2019, CDC, local and state public health partners, the U.S. Department of Agriculture (USDA), and the Food and Drug Administration (FDA) investigated a multistate outbreak of 356 *Salmonella* Reading infections from 42 states and the District of Columbia (DC) linked to turkey. The outbreak strain was isolated from raw turkey products, raw turkey pet food, and live turkeys. In July 2018, CDC and USDA’s Food Safety and Inspection Service (FSIS) shared outbreak investigation results with representatives from the U.S. turkey industry, engaging with an industry group rather than a specific company for the first time during an outbreak, and CDC issued a public investigation notice. During the investigation, four recalls of turkey products were issued. Evidence suggested that the outbreak strain of *Salmonella* was widespread in the turkey industry, and therefore, interventions should target all parts of the supply chain, including slaughter and processing facilities and upstream farm sources.

## Epidemiologic Investigation

In January 2018, through routine state surveillance, Minnesota Department of Health investigators identified four *Salmonella* Reading infections with an indistinguishable pulsed-field gel electrophoresis (PFGE) pattern, suggesting they likely shared a common source. One patient had consumed ground turkey, and two lived in the same household where pets in the home ate raw turkey pet food. Minnesota investigators also identified this same strain in one sample of retail ground turkey. This PFGE pattern is the most common subtype of *Salmonella* Reading; however, the Reading serotype is uncommon, not ranking in the 20 most common types of human *Salmonella* infections reported in the United States (*1*). In response to Minnesota’s investigation, PulseNet,* the national laboratory network for foodborne disease surveillance, was queried for additional *Salmonella* infections with this PFGE pattern. CDC began a multistate cluster investigation, collecting information on patient exposures from local and state health departments and information on food and pet food products from FDA and FSIS.

CDC defined a case as an infection with *Salmonella* Reading with the outbreak PFGE pattern with illness onset from during November 20, 2017–March 31, 2019. Patients were interviewed to collect information on consumption of turkey and other poultry foods, exposure to raw poultry pet food, and contact with live poultry.

Investigators from DC Health and the Iowa Department of Health identified two illness subclusters of cases in which attendees ate at a common event before becoming ill. The two events occurred in November 2018 and February 2019, and 152 persons became ill, including 51 whose clinical isolates matched the outbreak strain and 101 who had clinically compatible illness without culture confirmation of *Salmonella* infection. Investigators identified whole turkey and boneless roast turkey as the food items significantly associated with illness at these two events and found that turkey was not handled or prepared in accordance with FSIS guidelines and was not held at proper temperatures to prevent bacterial growth (*2*).

**FIGURE 1:**
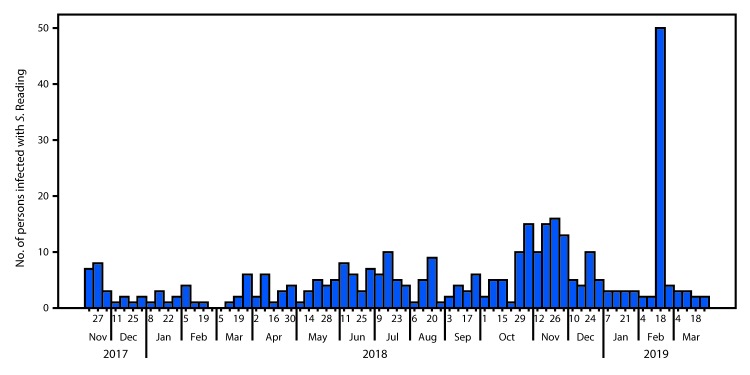
Number of persons (N = 356) infected with the outbreak strain of *Salmonella* Reading by date of illness onset* — United States, November 20, 2017–March 31, 2019 * Approximately 20% of illness onset dates were estimated from other reported information.

**FIGURE 2:**
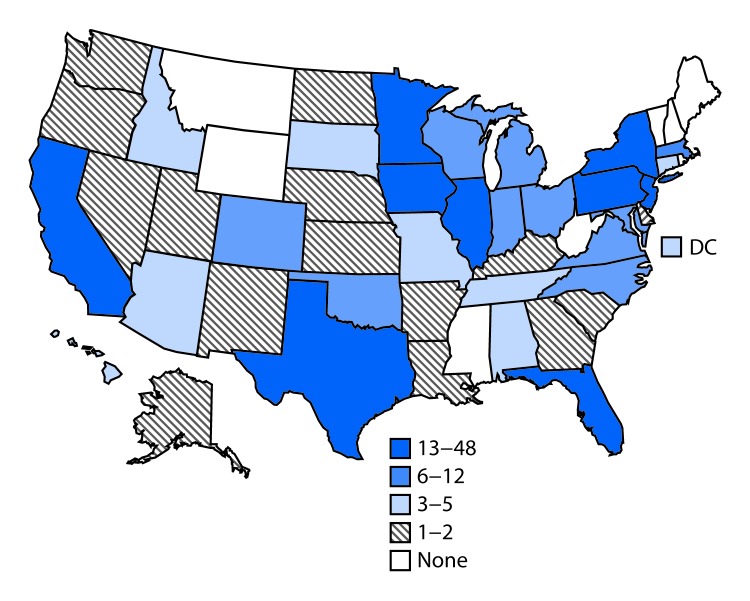
Number of persons (N = 356) infected with the outbreak strain of *Salmonella* Reading, by state — United States, November 2017–March 2019 **Abbreviation:** DC = District of Columbia.

Overall, 356 outbreak cases from 42 states and DC were identified (Figure 1) (Figure 2). Patients ranged in age from <1 to 101 years (median = 42 years), and 175 (52%) of 336 patients for whom information on sex was available were male. Among 300 patients with available information, 132 (44%) were hospitalized, and one died. Among 198 interviewed patients, 132 (67%) reported direct or indirect contact with turkey in the week before illness; 123 reported preparing or eating turkey products that were purchased raw (including whole turkey, turkey pieces, and ground turkey), four became sick after pets in their home ate raw ground turkey pet food, and five worked in a facility that raises or processes turkeys or lived with someone who worked in such a facility. No common type, brand, or source of turkey was identified.

## Product Testing and Laboratory Investigation

During the investigation, the outbreak strain was identified in 178 samples of raw turkey products from 24 slaughter and 14 processing establishments in 21 states that were collected by FSIS as part of routine testing and in 120 retail turkey samples collected as part of the National Antimicrobial Resistance Monitoring System retail meat sampling program (*3*,*4*). These samples represented several brands and types of raw turkey products. The outbreak strain was also identified in 10 samples from live turkeys in several states.

Investigators from the Arizona State Public Health Laboratory and the Michigan Department of Agriculture and Rural Development identified the outbreak strain in two of three unopened ground turkey samples collected from two patient homes. These were the same brand of ground turkey but were produced in different facilities. Investigators from the Minnesota Department of Agriculture identified the outbreak strain in samples of two brands of raw turkey pet food that were served to pets in patients’ homes. No commercial connections or common source materials were identified among any of these facilities.

## Public Health Response

In July 2018, CDC and FSIS shared investigation results with the National Turkey Federation, an industry group that represents turkey farmers and processors, and asked about steps they could take to reduce *Salmonella* contamination in their products. This was the first time that CDC and FSIS engaged an industry group rather than a specific company during an outbreak, a step taken because no single product or common supplier was identified. Upon learning of the outbreak, the National Turkey Federation compiled *Salmonella* control programs to share industry-wide, conducted studies about *Salmonella* in processing plants, sought research on interventions, and began bolstering consumer food safety education (*5*).

On July 19, 2018, CDC issued an initial investigation notice describing the outbreak. Because no single, common supplier of turkey products was identified, CDC reminded consumers to always follow appropriate food handling techniques to prevent *Salmonella* infection (*6*).

Four recalls of turkey products were issued during this investigation after investigators identified the outbreak strain in ground turkey and raw turkey pet food associated with illnesses in Arizona, Michigan, and Minnesota. In February 2018 and January 2019, approximately 4,000 pounds of raw turkey pet food were recalled by two separate pet food companies based in Minnesota. In addition, in November and December 2018, approximately 300,000 pounds of ground turkey products were recalled by two turkey establishments of the same company.

## Discussion

From 2018 to 2019, public health officials investigated a large and protracted multistate outbreak of *Salmonella* infections linked to raw turkey products. Evidence demonstrated that the outbreak strain was present throughout the turkey industry in live turkeys and in raw turkey products meant for human and animal consumption. This was one of the first times CDC used a new communication tool, an investigation notice, to provide information and recommendations to consumers when no specific product source was identified during an outbreak investigation (*6*). This tool allows for timely and pertinent communication with partners, which is important to identifying the cause of outbreaks and stopping them more quickly.

Although previous multistate outbreaks of *Salmonella* Heidelberg (associated with ground turkey) (*7*) and *Salmonella* Hadar infections (associated with turkey burgers) (*8*) have occurred, a noteworthy aspect of this outbreak was that no single common source or supplier was identified as the cause of illnesses. For this investigation, it was necessary to determine whether illnesses were part of an outbreak or sporadic infections with a common strain of *Salmonella*. The evidence suggested that an outbreak occurred and that turkey products were the source for two reasons. First, there was a strong epidemiologic association between illness and exposure to turkey. Second, laboratory evidence indicated that the outbreak strain was present in turkey facilities around the country and in live turkeys. The outbreak strain might have been introduced into the turkey supply chain and subsequently spread to many establishments and products throughout the industry before isolates from the Minnesota investigation were identified and the number of isolates were enough to initiate a multistate investigation.

Because contamination was widespread, interventions needed to target all parts of the supply chain, including slaughter and processing facilities as well as upstream farm sources. Although elimination of *Salmonella* from poultry flocks and products is challenging, the responsibility to develop effective strategies for *Salmonella* reduction along the production chain begins with industry. This investigation ended in April 2019 because new cases of illness decreased; however, cases continue to be identified. Evidence suggests that this outbreak strain has become widespread within the turkey production industry, warranting continued preventive actions to reduce contamination.

The two illness subclusters in this outbreak indicate improper handling and cooking of raw turkey products and highlight the need to reinforce consumer education. A 2017 study found that adherence to food safety practices among persons preparing turkey burgers was low but did improve after watching a USDA video on proper thermometer use (*9*). This same study also found very low adherence to CDC’s recommended steps for handwashing during food preparation and noted that approximately half of the participants contaminated other kitchen items, such as spice containers, by touching them while preparing turkey (*9*). These findings underscore the impact that food safety messaging can have on consumer behavior and the importance of proper food safety throughout the food preparation process. Consumers should always thaw turkeys safely (in the refrigerator in a container, in a leak-proof plastic bag in a sink of cold water, or in a microwave oven following the manufacturer’s instructions), avoid the spread of bacteria from raw turkey by keeping it separate from other foods and keeping food surfaces clean, and cook turkey to 165°F (74°C), measured on a food thermometer inserted into the thickest portions of the breast, thigh, and wing joint.^†^ In addition to emphasizing the importance of food safety messaging, this outbreak reinforced the need for awareness of the recommendations against feeding pets a raw meat diet, which can lead to both human and animal illnesses (*10*). Finally, industries can take steps to provide consumer education through their marketing programs and on product packages. Consumers, public health agencies, and industry officials all play important roles in promoting and implementing *Salmonella* prevention and control strategies to prevent future illnesses.

**Summary**

**What is already known about this topic?**

*Salmonella* Reading is a serotype that is uncommonly associated with human illness. *Salmonella* outbreaks have previously been associated with ground turkey and turkey burgers.

**What is added by this report?**

During November 2017–March 2019, a multistate outbreak of *S.* Reading involving 356 cases in 42 states occurred. Patients reported exposure to various turkey products, suggesting industry-wide contamination, a novel type of outbreak in which contamination is not isolated to a single food or facility.

**What are the implications for public health practice?**

Interventions should target all parts of the supply chain, including slaughter and processing facilities and upstream farm sources. Public health agencies and industry can take steps to provide more consumer education about food safety.

## Footnotes

* https://www.cdc.gov/pulsenet.

† https://www.cdc.gov/foodsafety/communication/holiday-turkey.html.
